# Drug-Loaded Biomimetic Ceramics for Tissue Engineering

**DOI:** 10.3390/pharmaceutics10040272

**Published:** 2018-12-13

**Authors:** Patricia Diaz-Rodriguez, Mirian Sánchez, Mariana Landin

**Affiliations:** 1 Departamento de Farmacología, Farmacia y Tecnología Farmacéutica, Facultad de Farmacia, Universidade de Santiago de Compostela, 15782 Santiago de Compostela, Spain; mirian.sanchez.fernandez@usc.es (M.S.); m.landin@usc.es (M.L.); 2 Instituto de Bioingeniería en Red Para el Envejecimiento Saludable-IBEROS Network, 15782 Santiago de Compostela, Spain

**Keywords:** bioceramics, biomimetic scaffolds, tissue engineering, bone, local drug delivery

## Abstract

The mimesis of biological systems has been demonstrated to be an adequate approach to obtain tissue engineering scaffolds able to promote cell attachment, proliferation, and differentiation abilities similar to those of autologous tissues. Bioceramics are commonly used for this purpose due to their similarities to the mineral component of hard tissues as bone. Furthermore, biomimetic scaffolds are frequently loaded with diverse therapeutic molecules to enhance their biological performance, leading to final products with advanced functionalities. In this review, we aim to describe the already developed bioceramic-based biomimetic systems for drug loading and local controlled release. We will discuss the mechanisms used for the inclusion of therapeutic molecules on the designed systems, paying special attention to the identification of critical parameters that modulate drug loading and release kinetics on these scaffolds.

## 1. Introduction

The aging of the population has led to the need for a new set of engineered biofunctional systems, designed not only to restore the functionality of diseased tissues, but to improve patient’s quality of life. Those advanced systems usually present complex design and chemical compositions aiming to regenerate damaged structures by, in most cases, combining biomaterials, cells, and therapeutic molecules. In this context, the tissue engineering field is focused on the development of biological substitutes able to restore, maintain, or improve tissue function or a whole organ [1].

Biomaterials, “implantable materials that perform their function in contact with living tissues”, are one of the pillars of the tissue engineering field [2]. They are commonly used to replace and restore the function of damaged tissues, and must fulfill a set of requirements, such as biocompatibility and mechanical stability, to ensure adequate performance [3,4]. There are four main families of biomaterials that can be used as tissue engineering scaffolds, classified based on their chemical composition as metals, ceramics, polymers, and composites (composed by a mixture of two of the above mentioned types).

Metals are generally used as permanent implants for load-bearing applications. This group includes titanium and its alloys, stainless steel, and cobalt–chromium alloys, as the most commonly used examples. The group of ceramics includes a wide variety of materials generally categorized in two groups: bioinert and bioactive. Finally, polymers can be obtained from natural origin or synthetic source, and contain a big set of molecules with variable properties depending on their chemical structure and molecular weight, among others [5].

Every family of biomaterials has its own benefits and drawbacks. While ceramics are biocompatible and can be bioactive and bioresorbable, their mechanical properties are not the best, due to their brittle character. On the other hand, metals present optimal mechanical properties, but they suffer of corrosion and toxicity problems. Furthermore, polymers present variable properties dependent on their chemical structure, but they generally lack of bioactivity [2]. Bioceramics, biocompatible ceramic materials applicable for biomedical or clinical uses, are one of the most commonly used materials for the skeletal system regeneration (bones, joints, and teeth) due to their chemical and physical properties [6,7]. The use of bioceramics for clinical application is relatively new and appeared in the late 1960s, as an alternative to improve hard tissue replacements and some biocompatibility problems associated with metallic implants [8,9].

Bone is characterized by a highly organized hierarchical anisotropic structure, from the nano to the macro scale, responsible for its unique mechanical behavior with remarkable strength and toughness. From the chemical point of view, bone is considered a nanocomposite of an inorganic phase nanocrystalline carbonated apatite and an organic phase (collagen, glycoproteins, and mucopolysaccharides). These bone features allow for the body mobility, support, and protection of the organs [10,11,12,13]. Furthermore, bone is the second most transplanted tissue, just after blood, and despite the huge advances on the development of biomaterials able to restore or replace it, no perfect materials has been obtained [9,14].

Biomimetic approaches are widely used to develop scaffolds for tissue engineering applications as a strategy to improve systems performance. The design of these scaffolds is commonly based on the mimesis of either the native tissue structure and/or composition of the material to be replaced [12].

### 1.1. Ceramics and Their Applications in Biomedicine

Ceramic materials have had a great expansion over the centuries, evolving from their use as simple pottery to their current application in the repair and reconstruction of damaged or diseased parts of the body, by the so-called bioceramics [6]. Even though the term ceramics includes a wide variety of inorganic nonmetallic materials, they present common characteristics as hard refraction, polycrystallinity, difficulty to shear plastically, high melting temperatures, low electric conductivity, and corrosion resistance [7,15].

These properties made them suitable for their application in hard tissue replacement, being currently used in periodontal, cranial, maxillofacial, dental, middle ear, spinal, and otolaryngology surgery [9]. Bioceramics are generally categorized into two main groups considering their biological performance: bioinert or bioactive, as described in Table 1. Bioinert ceramics constituted the first generation of bioceramics and were employed in femoral heads of hip prostheses. This family of biomaterials includes alumina, zirconia, pyrolytic carbon, and silicon carbides. Among this group, alumina and zirconia are the most commonly used. They are typically used for load-bearing applications, due to their excellent mechanical properties with high fracture toughness, wear resistance, strength, and fatigue resistance [3,16,17].

**Figure 1:**
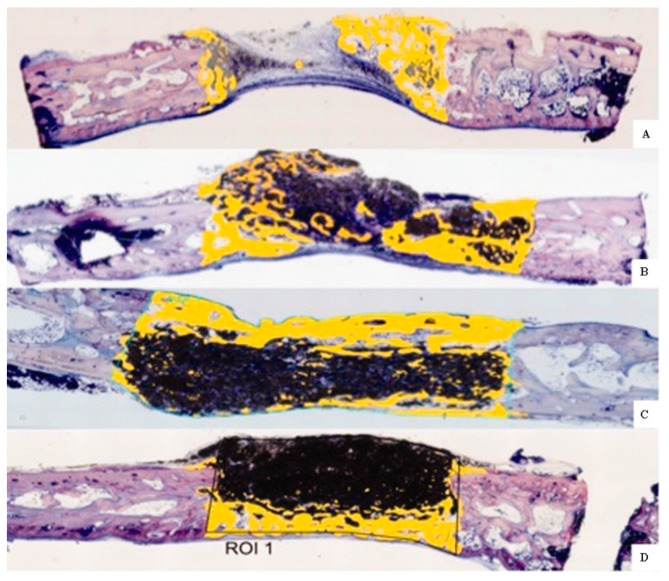
Histological cross-sections after 60 days of implantation for each of the three HA/β-TCP/collagen scaffolds: control (**A**), 40/30/30 scaffolds (**B**), 50/20/30 scaffolds (**C**), 60/20/20 scaffolds (**D**). Black: residual scaffold. Yellow: bone remodeling site (resorption foci). Reprinted from [25] with permission from Wiley.

The search for biomaterials able to integrate into the body without promoting a fibrous capsule formation has led to the development of bioactive ceramics—a second generation of bioceramics. This group of bioceramics includes calcium phosphates, bioactive glasses, aluminum oxides, iron calcium oxides, coralline, zinc calcium phosphorous oxides, and zinc sulphates calcium oxides. Calcium phosphates are the most widely used bioactive ceramics due to their similarities to the mineral phase of bone. There are different types of calcium phosphate ceramics with variable Ca/P ratios and crystal phases that modulate ceramic degradation. Hydroxyapatite (Ca_10_(PO_4_)_6_(OH)_2_; HAp) is the least soluble form of calcium phosphate, a characteristic that limits its bioactivity and application in the tissue engineering field. Tricalcium phosphate (Ca_3_(PO_4_)_2_; TCP) presents a lower Ca/P ratio and two crystalline forms (α-TCP and β-TCP) with variable solubility, among them β-TCP has the lowest dissolution rate. β-TCP is reabsorbed, in vivo, and replaced by remodeled bone in approximately 13–20 weeks after implantation [19,20]. However, the relatively high solubility of β-TCP compromises the mechanical support of the scaffold during the required time. To counterbalance the drawbacks of both β-TCP and HAp, composite ceramics formed by both components, denominated biphasic calcium phosphates (BCP), are commonly used. BCP present better mechanical properties than β-TCP, together with improved bone ingrowth when compared to their single components [21]. Figure 1 shows the bone tissue growth and degradation of HAp/β-TCP/collagen scaffolds with variable proportions of HAp/β-TCP after 60 days implantation on rabbit calvarial defects. An increase in the HAp proportion leads to a decrease in the biomaterial resorption rate [25]. Calcium phosphates are widely applied ceramics in the field of bone regeneration as fillers, tissue engineering scaffolds, and bioactive coatings and composites, due to their excellent biocompatibility, osteoconductive properties and similarity to native bone [26].

Bioactive glasses are the second most important family of bioactive ceramics. These materials are able to promote the formation of a hydroxyapatite-like layer when immersed in biological fluids in few hours, and show a strong bond to surrounding bone tissue [19]. Bioactive glasses are used for numerous applications, such as middle ear small bone replacement, cochlear implants, endosseous ridge maintenance implants, jaw bone defects fillers… [22]. They present a composition based on SiO_2_, CaO, P_2_O, and NaO which, in addition to being essential to guarantee the regeneration of tissues, endows them with antibacterial properties [23,24]. Ion release obtained during their resorption activates cell differentiation by the activation of specific genes [27], and also has devastating effects for some bacterial species [23,24]. The ability of bioactive glasses to form a hydroxycarbonate apatite layer in aqueous media is not only used to design and develop scaffolds for tissue engineering, but also to protect enamel during tooth bleaching treatment [28]. 

As for corals, they are mainly composed by calcium carbonate (CaCO_3_), and have a unique interconnected porosity conditioned by the origin species. Their highly porous structure closely resembles the trabecular bone morphology, and facilitates reabsorption without inflammatory responses. Moreover, coral can be transformed into HAp by a hydrothermal exchange retaining the original structure [19].

**Figure 2:**
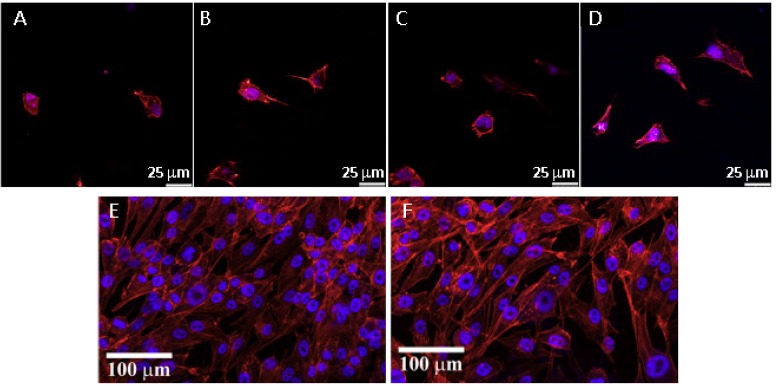
Fluorescence microscope images of adipose-derived mesenchymal stem cells cultured for 6 h on HAp with variable surface topography; control (**A**), nanosheet (**B**), nanorod (**C**) and micro–nanohybrid (**D**). MG63 human osteoblasts cultured for 15 days on zirconia (**E**) or alumina–zirconia particulate composites (**F**). Actin filaments are stained in red, while the cell nuclei are stained in blue. Adapted from [30,35] with permission from Elsevier.

The assessment of the biological performance of bioceramics for tissue engineering follows the same criteria as for any other material. The interaction with the native tissue is commonly studied initially, *in vitro*, in culture, with relevant cell types, before *in vivo* evaluation. As most of the designed bioceramic scaffolds are prepared to be used for bone tissue engineering, the test of cell performance (usually osteoconductivity) is commonly evaluated using mesenchymal stem cells (MSCs) and/or osteoprogenitor cells. These studies usually analyze cell morphology, viability, proliferation, and differentiation when cultured on or in contact with the scaffolds, together with extracellular matrix mineralization [29,30,31,32]. In general terms, osteoblasts and MSCs usually attach and colonize the developed bioceramics, promoting their bond to surrounding tissue. Figure 2 shows the morphology of MSCs cultured on HAp with variable topography, as well as osteoblasts cultured on zirconia (E) or alumina–zirconia particulate composites. Moreover, the bioactivity of scaffolds is traditionally assessed by their incubation in simulated body fluid. A biomaterial is considered bioactive when an apatite layer is formed after incubation [33,34].

### 1.2. From Conventional Ceramics to Biomimetic Systems

The exploration for biomaterials with improved biological properties, in the case of bone tissue ensuring osteoinduction, osteoconduction, and osseointegration, has led to the development of bioceramics aiming at the mimesis of biological systems. The need of biomimetic structures comes from the recognized effect of scaffold structure on bioactivity and osteoinduction. Furthermore, the possibility to develop novel systems able to be recognized by osteoclasts as autologous bone and, therefore, being part of the physiological bone remodeling, could represent a huge advance in the tissue engineering field. In this context, biomaterial parameters as nanotopography, pore size, porosity, and interconnectivity are related to scaffold resorption, mineralization, and vascularization [25,35,36]. It is well known that an adequate interconnected porous structure is essential to guarantee cell colonization and tissue formation. Moreover, pore size and total scaffold porosity drastically condition cell mechanical properties and cell attachment. Macropores (pore diameter >100 µm), are required for cell penetration and vascularization while micropores (pore diameter 0.1–10 µm) improved cell attachment by creating a rough surfaces and facilitating the penetration of body fluids [21,37]. On the other hand, nanopores (pore diameter <0.1 µm) increase the specific surface area of the scaffolds, allowing for drug loading and higher bioactivity in the case of bioactive materials. 

**Figure 3:**
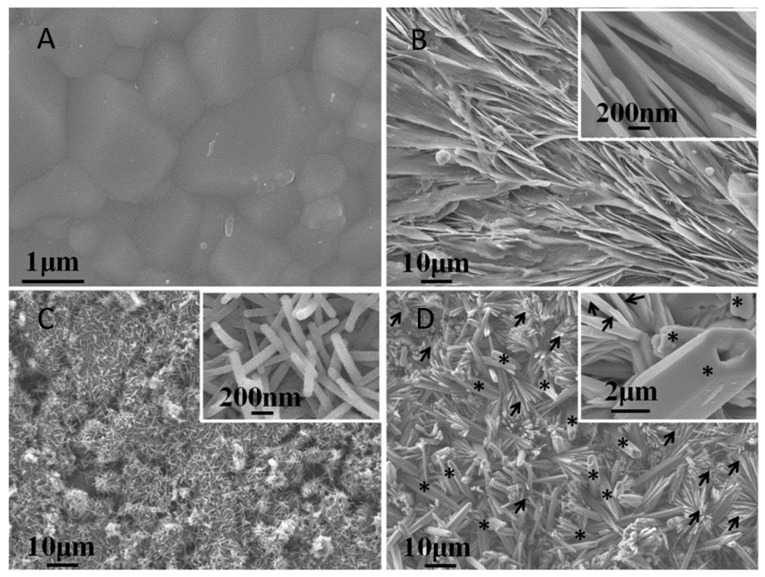
Difference in surface topography of standard TCP (**A**) when compared to biomimetic surfaces with variable topography; nanosheet (**B**), nanorod (**C**), and micro–nanohybrid (**D**). Reprinted from [39] with permission from ACS.

The mimesis of micro/nano topography, to imitate natural bone in metals, polymers, and ceramics, has been used as an approach to accelerate cell attachment, migration, proliferation, and differentiation, leading to an enhancement of tissue integration and improved biocompatibility [38,39]. As shown in Figure 3, the synthesis of biomimetic ceramic surfaces led to the production of materials with complex nanotopographies that increase osteoblast attachment, spreading, adhesion, proliferation, and osteogenic differentiation [39]. Furthermore, the synthesis of biomaterials with a composition similar to the tissue to be replaced is a commonly used strategy to obtain biomimetic scaffolds. Bone is a composite material itself, consisting of an organic phase and a mineral phase. Therefore, numerous composite biomaterials have been developed, aimed at mimicking tissue chemical and mechanical properties [40,41].

The development of biomimetic scaffolds is not only used for the repair of bone defects, but also can be used to restore other musculoskeletal tissues as cartilage. For these applications, polymeric systems are commonly used to recapitulate the physicochemical features of the tissues. Moreover, these systems not only allow for a mimesis of the tissue but also control immune responses to implantable materials [42].

**Figure 4:**
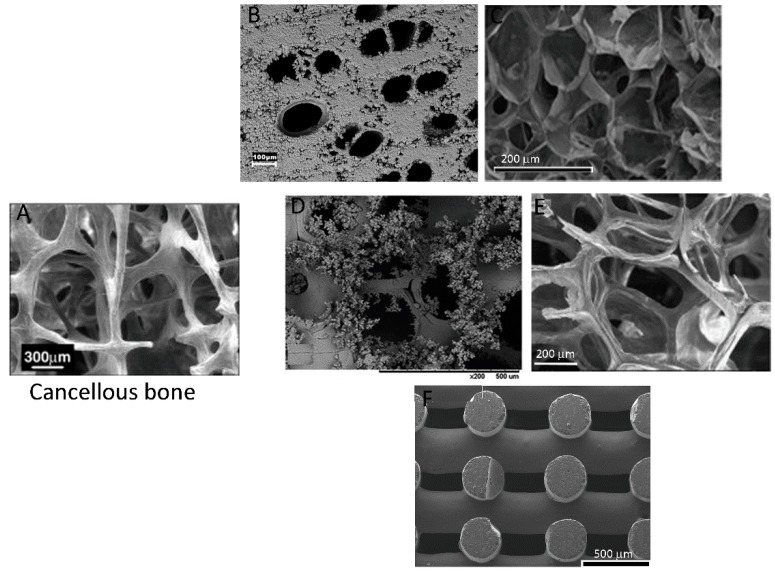
Scanning electron microscopy images of bone structure (**A**), Reproduced with permission from [50], published by Elsevier, 2007) in comparison to biomimetic bioceramics obtained from variable techniques: (**B**) bioinert silicon carbide using a natural precursor mold; (**C**) hydrogel-bioceramic composites of gelatin-3-(4-hydroxyphenyl) propionic acid (Gtn-HPA)/carboxymethyl cellulose-tyramine (CMC-Tyr), and fish-derived calcium phosphate, obtained by foaming. Reproduced with permission from [51], published by Wiley, 2018; (**D**) Alumina–zirconia porous ceramics coated with HAp after soaking with SFB for 7 days. Reproduced with permission from [43], published by Wiley, 2018; (**E**) biomimetic mesoporous bioactive glasses obtained by precursor transformation. Reproduced with permission from [46], published by Elsevier, 2008; (**F**) bioactive glass scaffolds obtained by additive manufacturing. Reproduced with permission from [48], published by Elsevier, 2016.

Different strategies can be employed to obtain biomimetic ceramics and/or bioceramic-based biomimetic composites. The most commonly used strategies as well as the morphology obtained when compared to cancellous bone are described in Figure 4. The manufacture of biomimetic-bioinert ceramics usually requires a natural precursor mold that will be then transformed to a ceramic complex structure (Figure 4B) [15]. However, other techniques, such as foaming, can also be used to develop, for example, alumina–zirconia scaffolds [43].

In the case of bioactive ceramic-based scaffolds, they can be obtained by numerous methods, such as extraction from natural bone, molecule-directing mineralization, foaming (Figure 4C), salt leaching, simulated body fluid (SBF)-induced calcium phosphate formation (commonly described as biomineralization) (Figure 4D), precursor transformation (Figure 4E), and hydrothermal reaction [26,43,44,45,46]. The development of advanced manufacture techniques, such as additive manufacturing, has opened a new set of possibilities for the synthesis of bioactive and biomimetic ceramics (Figure 4F). This approach allows rapid scaffold synthesis with tunable mechanical properties and a porosity gradient closer to resemble the physiological structure of bone [47,48,49].

#### 1.2.1. Biomimetic Scaffolds Based on Inert Bioceramics

Other types of biomimetic ceramics are the ones developed based on the natural structure of biological systems such as wood or cellulose. These systems are based on the use of the natural interconnected hierarchical porous structure of the mold material to obtain ceramics with the desired interconnectivity and porosity. In this context, one of the most commonly used ceramics, due to their excellent mechanical properties, is biomorphic silicon carbide ceramics (BioSiC). The synthesis process of this ceramic starts with the pyrolization of the precursor to obtain carbon that is then infiltrated with silicon to obtain 3D porous structures formed by silicon carbide crystals. These systems have shown adequate compatibility in physiological fluids, for example, with blood components [18,52]. One step forward in the use of these ceramics is the development of composite systems made by a collagen/hydroxyapatite core and a BioSiC shell to obtain biphasic bone-mimicking scaffolds. These systems have the ability to promote new bone formation in vivo, that is increased when mesenchymal stem cells (MSC) are added during implantation [53]. However, it is necessary to point out the lack of resorption of these ceramics when implanted.

#### 1.2.2. Biomimetic Scaffolds Based on Calcium Phosphate

As stated above, calcium phosphates are widely used ceramics in the field of bone regeneration, due to their osteoinduction ability and mimesis of tissue composition. However, calcium phosphate ceramics are characterized by their low mechanical strength and their brittleness, which limits their use in long bone repair. Table 2 presents several approaches taken to overcome this problem that have also translated into other benefits.

A common alternative has been to include calcium phosphate particles into different networks with advanced mechanical properties, such as natural rubber latex [54]. Another alternative is the synthesis of biomimetic protein–ceramic composites, using the cell derived extracellular matrix as the protein component and biphasic calcium phosphate as the ceramic component. These systems were able to promote the osteogenic differentiation of MSCs with good in vivo performance and no immunogenic response [55].

Composite systems of calcium phosphates and metals like TiO_2_ (titania) have also been developed in order to counterbalance the poor mechanical properties of the ceramic component. The addition of the metal has allowed obtaining systems with enhanced compression strength and improved sintering characteristics [20,56]. Furthermore, these systems are able to maintain the bioactivity of calcium phosphate ceramics, promoting the proliferation and their in vitro colonization by osteoblasts [20].

The use of 3D printing has also been explored to obtain polymer–ceramic composites for osteochondral regeneration. These systems were based on the combination of a synthetic polymer, Polyethylene Glycol (PEG), with the ceramic β-TCP showing improved interfacial integration and adequate mechanical characteristics [57]. In a similar way, the use of hierarchical biomimetic HAp ceramic surfaces at the micro- and nanoscale has shown to promote cell attachment and proliferation, and serve as an osteogenic stimulus for adipose-derived stem cells, both in vitro and in vivo, by the activation of the Akt signaling pathway. Furthermore, these materials promoted an increase in the secretion of pro-angiogenic factors from differentiated cells [35].

The search for 3D structures able to resemble complex tissue characteristics has led to the development of ceramic systems not only focused on the mimesis of porosity and composition, but also on the physiological capillary effect, taking advantage of interconnected gradient channels able to mimic bone network. These scaffolds, made by HAp, also have the capacity to be homogenously self-seeded [58]. Using a similar approach, the combination of synthetic polymers, such as PEG with HAp, by pressure-dependent polymer agglomeration and vaporization, was able to recreate the nanoscale transport mechanisms of nature, and this hierarchical structure allowed for the survival and growth of seeded cells [59].

Other calcium phosphate ceramics than those mentioned above can also be used for the development of biomimetic composite systems. One example is the use of fish-derived calcium phosphate, that has provided the mechanical properties of a gelatin and carboxymethyl cellulose hydrogel [51]. Furthermore, the synthesis of highly carbonated HAp has been hypothesized to better resemble the crystallinity of natural HAp, showing excellent results of resorption and biocompatibility, while their functions are modulated by the degree of carbonation [60]. Furthermore, calcium carbonate nanoparticles can be used for cell-targeted delivery of drugs, genes, and proteins. In these applications, the morphology and size of the nanoparticles modulate the performance of the systems [26].

Recent studies have been focused not only on evaluating the mesenchymal stem cell response to biomimetic ceramics, but also to elucidate their behavior when these biomaterials are subjected to osteoclasts precursor cells, as osteoclasts are not only implicated in material resorption, but also in osteoblast activity. In this context, the development of micro-nanostructured biomimetic calcium phosphate substrates hampered the resorption activity of osteoclasts precursor cells when compared to bulk materials, pointing to the role of topography on osteoclast formation and activity [61].

#### 1.2.3. Biomimetic Scaffolds Based on Bioactive Glasses

Bioactive glasses are a family of ceramics widely used in bone regeneration, due to their ability to promote hydroxyapatite formation and their osteoconductive character. These characteristics make them suitable to be incorporated as the inorganic component on collagen/gelatin scaffolds, acting as mineralization agents with osteoconductive properties, and improving the poor mechanical strength of those hydrogels [62,63]. Moreover, bioactive glasses are commonly used to promote bioactivity when added to inert systems, such as methacrylate-based dental adhesives, to increase the bond strength between the tissue and the adhesive [64,65]. Their incorporation is able to increase the mineralization of other polymeric systems, such as polyacrylic acid and chitosan [40,41].

Nanoparticles of bioactive glasses are also combined with chitosan membranes to promote biomineralization while preventing the invasion of periodontal defects by soft tissues [66]. In a similar way, the inclusion of bioactive glasses on resins is able to improve the mechanical properties of the resins, increasing the mineralization of the systems [67]. The combination of macroporous bioactive glasses with inert polymers, such as polycaprolactone (PCL), obtained by additive manufacturing, improved the mechanical properties of both components. The coating of these scaffolds with collagen–HAp promoted high osteoconductivity [4].

## 2. Biomimetic Ceramics as Drug Delivery Platforms

As described previously, biomimetic systems are those designed to reassemble the structure, and biophysical and mechanical properties of the native tissue. These systems are intended to behave as temporary matrices for cell attachment, proliferation, differentiation, and extracellular matrix deposition [39]. To improve their performance even further, therapeutic molecules can be incorporated. The drug loading of scaffolds aims at promoting biologic repair, providing support for treatment and decreasing postsurgical complications. Several studies, including antibiotics, growth factors, antitumorals, or antiresorptive drugs, have been carried out in the field of bone tissue engineering, particularly using biomimetic ceramics [68]. Despite the huge efforts on treating chronic osteomyelitis, the reconstruction of the tissue after debridement continues to be a challenge, due to the difficulty with eradicating pathogens [2]. The synthesis of implantable scaffolds able to promote a long-term delivery of antibiotics, while enhancing tissue regeneration is one of the main goals of the recently developed drug-loaded biomimetic ceramics or ceramic composites.

On the other hand, growth factors are usually added to biomimetic ceramics to promote a specific cell response at the molecular level. The natural process of bone healing is controlled by a set of cytokines and growth factors involved in the recruitment of inflammatory cells in the acute phase, and MSCs at a later stage. MSCs should then proliferate and differentiate into osteogenic cells. Among all the biomolecules implicated in bone healing, bone morphogenic proteins (BMPs) play an essential role on bone repair, being responsible for the initiation of mesenchymal condensation. From all the BMPs family, particularly BMP-2, BMP-4, and BMP-7 have been studied for bone tissue engineering applications [69,70]. 

Vascularization is also a critical factor to ensure the clinical success of 3D scaffolds. These biomaterials should promote/allow the formation of new vessels able to provide oxygen and nutrients to attached cells, and function as routes to remove waste products. The incorporation of pro-angiogenic growth factors as vascular endothelial growth factor-A (VEGF) is a common strategy to promote vascularization on the developed scaffolds. Furthermore, VEGF also plays an important role in osteogenesis [71,72,73,74].

The local administration of drugs takes special interest when talking about chemotherapeutic agents to prevent recurrence in osteosarcoma patients after resection of the primary tumor. In those cases, the decrease of serious side effects when compared to systemic administration represents a huge improvement in the patient’s quality of life [12].

Bisphosphonates are anti-resorptive drugs that can be included to prevent bone mass loss by the inhibition of osteoclast function. They are commonly used to treat bone metabolism-related pathologies, such as osteoporosis, Paget’s disease, bone metastasis, and hypercalcemia [75]. Furthermore, the combination of BMP-2 with bisphosphonates has been found to have a synergistic effect on bone fracture repair [76].

### 2.1. Advantages of Using Biomimetic Ceramics for Controlled Delivery of Drugs

Biomimetic ceramics, or biomimetic ceramic composites, attempt to mimic the multidimensional and hierarchical structure of tissues. The incorporation of therapeutic molecules to those biomaterials should enhance their performance by ensuring tissue repair, treatment, and/or preventing post implantation pathologies or diseases by the controlled local delivery of loaded therapeutic agents [77]. The use of biomimetic ceramic scaffolds as platforms for drug release allow for an in situ drug delivery, decreasing the doses and the risk of systemic side effects. The local administration of therapeutic molecules could also help to reach a long-term effective drug concentration at the target site. Moreover, these platforms enable the administration of poorly water-soluble drugs without the need of any further pharmaceutical additives [9]. The possibility to incorporate therapeutic molecules within the structure of biomimetic bioactive ceramics allows for coupling drug release with ceramic resorption, that could be a tool to reach smart release profiles suitable to the needs of the tissue [77].

The increased surface area of biomimetic systems promote an enhanced protein adsorption, and could be used to load and controlled release therapeutic molecules [39]. Moreover, some biomimetic ceramics can be used as therapeutic molecules themselves. One example is bioactive glasses, that have demonstrated antibacterial activity against different bacterial strains [78]. The combination of both ceramic and drug therapeutic effects can lead to final biomaterials with enhanced biological activity.

### 2.2. Strategies for Biomimetic Ceramics Drug Loading and Release

**Figure 5:**
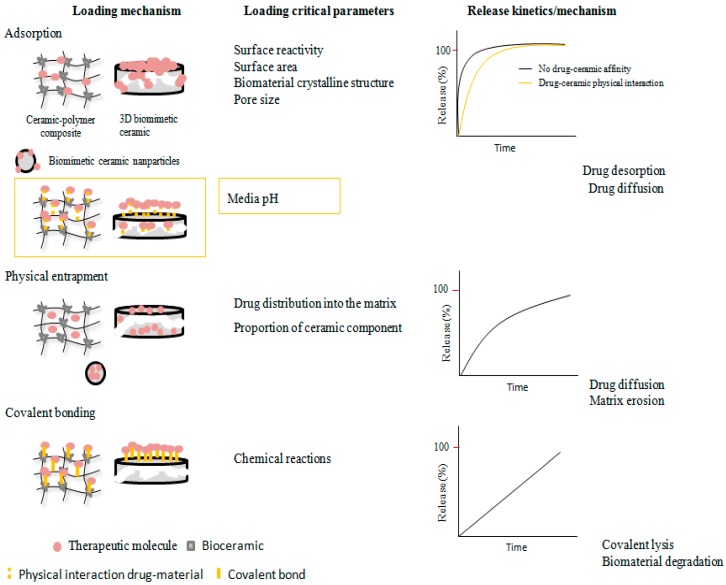
Summary of the main drug loading approaches used to incorporate therapeutic molecules on biomimetic ceramic systems.

The loading of biomimetic ceramics with therapeutic molecules can be carried out by different techniques categorized in the following groups: adsorption, physical entrapment, and covalent bonding. The main release profiles obtained, and the key parameters that condition the loading and release behavior, are summarized in Figure 5. Furthermore, Supplementary Table S1 presents the developed biomimetic bioceramic-based drug delivery systems.

#### 2.2.1. Adsorption

Adsorption is a superficial phenomenon and, as such, the surface characteristics of the biomaterials (reactivity and specific surface area) clearly condition the drug loading capacity and release [77]. The high scaffold-specific surface area allows the loading processes without the use of aggressive treatments, guaranteeing the stability of the therapeutic molecules. Their adsorption on biomimetic ceramics ensures a high loading capacity and a diffusion-controlled release. The increase in specific surface area (total surface per gram of ceramic) represents an augment on the surface available for drug adsorption and, therefore, the possibility to increase the amount of loaded drug. However, this increase in surface area also promotes a higher interaction with the physiological media that could increase the release rate and the resorption of the scaffold [79].

It has been hypothesized that the crystalline structure of ceramics, such as HAp, modulates its roughness and surface reactivity. Highly crystalline HAp powders promote a lower loading and a faster release of the anti-inflammatory drug, ibuprofen, than lower crystalline HAp [80].

Surface reactivity is related to the specific compound to be adsorbed and the loading solution selected. When the surface reactivity is low, the interaction between therapeutic molecules and the ceramic surface is weak, and the porous structure plays the main role on those processes [77]. On the other hand, when there is a physical interaction between therapeutic molecules and ceramic surfaces (van der Waals forces, hydrogen bonding, electrostatic or hydrophobic interactions) then surface reactivity also plays a role on the performance of the systems as drug delivery platforms. Except for hydrophobic interactions, they are all highly dependent on pH, modulating drug loading and release. pH has been used to control drug loading and release of proteins such as BMP-2 or VEGF, whose charges are dependent of the surrounding pH from TCP/bioactive glasses systems and gelatin/HAp composites, respectively [81,82,83]. Bisphosphonates have also been adsorbed into biomimetic ceramics, taking advantage of physical interactions. These compounds form strong ionic interactions with divalent ions, allowing for their efficient loading on calcium phosphate and bioactive glasses [84,85]. The presence of surface active groups in inert ceramics, such as biomorphic silicon carbide as well as in bioactive calcium phosphate and glass ceramics, allows for the adsorption of growth factors by electrostatic interactions, reaching a sustained drug release [86,87,88,89]. This affinity of calcium phosphate ceramics for proteins has been used to increase the drug loading and control release profile of collagen/HAp composites when compared to collagen hydrogels without the ceramic component [90].

The use of highly porous 3D systems with hierarchical structures loaded by an adsorption mechanism led to drug release profiles conditioned by the shapes and architectures of the biomimetic ceramic structures [26]. The porosity characteristics of biomaterials (total porosity, pore size, shape, and pore connectivity) modulate both drug loading and release kinetics. While an increase in total porosity tends to improve scaffold-loading capacity and speed, pore connectivity also plays a key role in the process. Low connectivity reduces both the loading rate and release rate. Pore size modulates the release profile obtained. Large pores allow for an initial fast release, while small pores serve as reservoirs of drug responsible for the long-term release. The ratio between large and small pores clearly modifies the release rate constant, that could be also modulated by controlling the connectivity and tortuosity of the pores [77,91,92].

Adsorption has also been used for biomimetic hierarchically microscale systems. In this context, the loading of strontium-doped amorphous calcium phosphate porous microspheres and zinc-doped HAp microspheres with therapeutic molecules (vancomycin or doxorubicin respectively) was able to promote adequate therapeutic effects. Those systems showed Higuchi release profiles, characteristics of systems whose release mechanism is controlled by drug desorption and diffusion [93,94]. Moreover, the addition of zinc to HAp promoted an enhancement on MSCs differentiation, and improved bone formation and healing [94].

Biomimetic apatite nanocrystals have also been used for drug loading and controlled release with variable applications. Their chemical composition, similar to the mineral component of bones, also endows them with good biodegradability and low toxicity when compared to silica, quantum dots, carbon nanotubes, or metallic magnetic particles, together with high stability and a pH-dependent dissolution rate [95,96]. These advantages over other nanosystems make HAp nanocrystals promising systems to be loaded with chemotherapy agents, and targeted to specific cell types of cancer treatment through their conjugation with specific moieties able to recognize tumor-associated surface markers [97]. In these cases, both the therapeutic agents and the functionalization molecules are adsorbed on the nanoparticle surfaces, taking advantage of the high surface area of the particles by a mechanism controlled by the concentration of the adsorbed molecule [98]. This approach is developed in order to promote the intercellular delivery of therapeutic molecules [96,99]. However, the reversible adsorption of therapeutic molecules by apatite nanocrystals also allows for a two-phase controlled release profile of the loaded molecules for up to two weeks [98].

Other ceramic-based nanosystems are hollow structured mesoporous carbon nanoparticles (HMNCs), and their structure mimics the morphology of red blood cells and aims at taking advantage of the surface area and pore volume of carbon for drug loading. These systems show a great ability to load and allow controlled release of aromatic drug molecules, due to their interaction with carbon though supramolecular π–stacking. These weak interactions allow for the stimuli-sensitive control release by modifying pH or applying ultrasound. Following these approaches, chemotherapeutic (doxorubicin)-loaded HMNCs have been developed, with a slow release at neutral pH and accelerated profiles at acidic environments. Furthermore, these systems were also responsive to ultrasound, allowing for controlling the release rate by modulating the irradiation power. These characteristics made them suitable for intratumoral drug delivery, allowing for an on-demand drug release profile [100]. Moreover, the use of supramolecular interactions has been described as a new approach to obtain biomaterials with hierarchically organized structures able to load and controlled release of different types of drugs [101].

The development of smart biomaterials does not only include the synthesis of materials with complex structures loaded with drug, but also the design of surfaces able to promote cell attachment by the incorporation of cell adhesion molecules, such as fibronectin and cadherin, to ceramic surfaces. These systems showed adequate osteoinduction and osteoconduction after in vitro testing with MSCs [102].

#### 2.2.2. Physical Entrapment

Therapeutic molecules can be trapped into the structure of bioceramics or bioceramic-based composites during their synthesis, or in subsequent processes. The homogenization of the drug inside the scaffold matrix is an important factor to avoid the burst release while drug release rate is conditioned by drug diffusion and scaffold resorption [13].

Composite systems of bioceramics/polymers are usually loaded with therapeutic molecules or model dies by their incorporation into the polymeric network. The degradation of the composite and the burst release from those systems depends on the proportion of the ceramic being between 5 and 10% (w/v) of calcium phosphate, the proper amount to reach the desirable drug release profile [51]. This strategy allows for zero-order release profiles controlled by a diffusion mechanism. 

In a similar way, the loading of collagen–HAp composite scaffolds with platelet-rich plasma, by soaking, promoted an increase on in vivo bone formation, while the incorporation of tetracycline by adsorption was able to promote a controlled release of the antibiotic [63]. 

The incorporation of drugs following this versatile approach also enables the inclusion of antibiotics such as the above mentioned tetracycline, or labile molecules such as the growth factor BMP-2, to bioceramic-based scaffolds, obtaining, in the case of BMP-2, systems with a controlled release of active protein for up to 21 days [103,104].

Biomimetic ceramics are also commonly used as coating systems for metals or polymers, to increase bone integration. In this context, drugs or model proteins are generally loaded in the coating layer by co-precipitation. The modulation of the addition time of the molecules during coating allows for a control over the release profile obtained. The addition of the model protein at the beginning of the coating leads to a two-stage release, while the addition at the end of the procedure promotes a burst release [105]. The incorporation of the therapeutic molecules into the ceramic coating layer, by a physical entrapment mechanism, promotes a higher loading capacity and a more controlled release [106].

Non-conventional drugs, such as strontium or copper, have also been included in biomimetic ceramics as therapeutic molecules. Strontium has been employed to dope calcium phosphate bone cements and amorphous calcium phosphate porous microspheres with beneficial effects against osteoporotic bone-weakening by promoting the osteogenic differentiation of MSCs, together with an inhibition of osteoclast activity [29,93]. The addition of copper to the glass matrix of composite biomimetic materials has been shown to encourage angiogenesis due to its ability to stimulate endothelial cell proliferation, also promoting osteoblast proliferation [62]. The inclusion of biological amounts of magnesium, carbonate, and strontium into calcium phosphate ceramics by ionic substitution, has been an approach to obtain ceramics with a closer chemical composition to the native bone. These co-substituted nanocrystalline apatites show stability and an adequate in vitro bioactivity [107]. The doping of calcium phosphate nanoparticles, with trace elements such as zinc, strontium, and silicon, embedded in a hydrogel mesh, have also been assessed for the treatment of osteoporosis [108]. 

Biomimetic mineralization of polymeric networks has been used not only to add standard HAp, but this technique can also be used to dope protein networks (collagen) with FeHAp, conferring magnetic properties to the developed systems. The incorporation of this property has allowed for controlling bone growth and improving in vitro scaffold performance [109]. The use of other advanced preparation techniques, such as freeze gelation of silica, allows for obtaining porous HAp ceramic scaffolds with tunable porosity. The mild conditions required for their synthesis represent a great advantage for the incorporation of labile molecules, such as proteins, into the ceramic structure. Moreover, the addition of proteins has the ability to improve the mechanical strength of the ceramics. In these systems, the characteristics of the proteins (solubility) strongly affect the magnitude of the release achieved under static conditions [110].

#### 2.2.3. Covalent Bonding

Despite physical adsorption being one of the most commonly used mechanisms to dope biomimetic ceramics with therapeutic molecules, this technique is usually not able to achieve a long-term retention of drugs, due to the weak interactions between biomaterials and therapeutic molecules. On the other hand, drug loading by physical entrapment or interaction by coating, electrodeposition, electrostatic spinning, layer by layer, requires a multistep approach that complicates the synthesis of the scaffolds. On this basis, the covalent bonding of therapeutic molecules, such as BMP-2-derived peptides, enables the achievement of zero-order kinetics for up to three months [111], avoiding the burst release and usually enhancing the stability of proteins [112].

The development of biphasic calcium phosphate-electrospun poly (l-lactide) scaffolds loaded with the adhesion peptide, RGD (Arg–Gly–Asp), by chemical conjugation, has allowed for obtaining scaffolds with enhanced cell adhesion and osteogenic differentiation with respect to unloaded scaffolds. The stable incorporation, by covalent bonding of RGD to the porous structures, promoted an increase in scaffold mineralization after 14 days [113].

## 3. Conclusions

The development of bioceramic-based systems, following biomimetic approaches, searches for the molecular, structural, and mechanical compatibility of the scaffolds. The incorporation of therapeutic molecules, cell attachment moieties, and/or ions to these systems further increases the performance of the scaffolds and, therefore, their potential applicability. However, despite the large number of published works directed at the development of biomimetic systems in tissue engineering, not many studies focus on its application as a drug administration platform capable of solving the problems associated with its use and meeting the needs of this emerging area. In addition, there are no clinical trials in progress, nor previous ones that have shown great experimental success. More efforts must be made to achieve an application and clinical use for the developed systems.

## Figures and Tables

**Table 1:** General classification of the main bioceramics used in the tissue engineering field based on their biological performance.

Bioinert	Strengths	Limitations	References
Alumina (Al_2_O_3_)	High fracture toughness, strength, and fatigue resistance	Not biodegradable	[16]
Zirconia (ZrO_2_)	Wear resistance	Risk of catastrophic fracture	[17]
Pyrolytic carbon	Biological inert, biocompatibility, hemocompatibility	Not biodegradable	[18]
Silicon carbide (SiC)	Excellent mechanical properties, biocompatibility	Not biodegradable	[15]
**Bioactive**	**Strengths**	**Limitations**	**References**
Calcium phosphates:	High biocompatibility, similarity to bone mineral phase	Brittle, poor mechanical properties	[19]
Hydroxyapatite (HAp)	Chemical composition and Ca/P ratio closer to bone than any other calcium phosphate	Low solubility, slow degradation rate	[19]
Tricalcium phosphate (TCP)	Crystalline forms of high solubility, resorbability	Low mechanical resistance, excessive resorbability	[20]
Biphasic calcium phosphates (BCP)	Improved bone growth over HAp and TCP alone	Hard to couple material degradation with tissue growth	[21]
Bioactive glasses	Strong bond to surrounding tissue, antibacterial properties	Poor mechanical properties	[22,23,24]
Coralline	Excellent porous structure, interconnectivity	High variability dependent on the source material	[19]

**Table 2:** Composites of calcium phosphate and several materials and benefits derived from these combinations as scaffolds in bone tissue engineering.

Biomaterial	Advantages	Reference
Calcium phosphate + natural rubber	Mechanical properties improvement	[54]
BCP + Extracellular Matrix proteins	MSCs osteogenic differentiation	[55]
	No immunogenic response	
BCP + TiO_2_	Mechanical properties improvement	[20,56]
	Compression strength	
	Proliferation improvement	
βTCP + Polyethylene Glycol (PEG)	Interfacial integration	[57]
HAp + PEG	Adequate osteogenesis, proliferation, cell attachment, and angiogenesis	[35]
3D printed HAp + PEG	Homogenous self-seeding	[58,59]
	Cell survival	
Fish calcium phosphate + gelatin/carboxymethylcellulose	Biocompatibility, resorption	[51]

## Supplementary Materials

The following are available online at http://www.mdpi.com/1999-4923/10/4/272/s1, Table S1: Summary of the developed drug loaded biomimetic bioceramics.
