# Supplementary material for: Drug-Loaded Biomimetic Ceramics for Tissue Engineering
Source: Pharmaceutics. 2018 Dec 13;10(4):272. doi: 10.3390/pharmaceutics10040272 (PMC6321415; doi:10.3390/pharmaceutics10040272)
Supplement: Supplementary file 1 [file pharmaceutics-10-00272-s001.pdf]

pharmaceutics

Supplementary Materials: Drug-Loaded Biomimetic Ceramics for Tissue Engineering

Patricia Diaz-Rodriguez, Mirian Sánchez and Mariana Landin

Table S1. Summary of the developed drug loaded biomimetic bioceramics.

Biomaterial	Loading technique	Drug	Release profile	Reference
HAp powder	Adsorption	Ibuprofen	Fickian diffusion	[80]
TCP/bioactive glasses	Adsorption	BMP-2	Initial burst followed by a prolonged release	[82]
Gelatin/HAp cryogel	Adsorption by microinjection	VEGF	Initial burst followed by a prolonged release	[83]
BoneSave granules (BCP)	Adsorption	Zoledronic acid	Initial burst followed by a prolonged release	[84]
Biomorphic silicon carbide(BioSiC)	Adsorption	VEGF	Initial burst followed by a prolonged release	[86]
HAp and α-TCP	Adsorption	BMP-2, bFGF	-	[87]
TCP/HAp granules.	Adsorption	BMP-2	Release for up to 14 days	[88]
HAp/Collagen nanocomposite fibers	Adsorption	BMP-2	Initial burst followed by a prolonged release	[89]
Collagen–HAp	Adsorption	Pamidronate, BMP-2	BMP-2 initial burst followed by a prolonged release	[90]
Biomorphic silicon carbide(BioSiC)	Adsorption	Vancomycin	Initial burst followed by a prolonged release	[91,92]
Mesoporous HAp microspheres	Adsorption	Doxorubicin hydrochloride	Two phase release controlled by pH	[94]
HAp nanoparticles	Adsorption	IgG	-	[96]
HAp nanoparticles	Adsorption	BMP-2	Two phase release profile	[98]
Nanocrystalline calcium phosphate apatite	Adsorption	Erythrosin	Two phase release profile	[99]
BCP	Adsorption	fibronectin/ cadherin	Two phase release profile	[102]
Gelatin-carboxymethyl cellulose/CaP	Physical entrapment	Dextran (model drug)	Zero order release	[51]
Alginate+nano-HAp/ collagen	Physical entrapment	BMP-2	Zero order release	[103]
Gelatin-HAp nanocomposites	Physical entrapment	Tetracycline	Zero order release	[104]
Calcium phosphate coatings of Ti	Physical entrapment	BSA (model protein)	Two phase release profile dependent on BSA content	[105]
Calcium phosphate coatings of Ti	Physical entrapment	BSA (model protein)	Release profile dependent on BSA incorporation technique	[106]
Apatitic bone cement	Physical entrapment	Strontium	Zero order release	[29]
Calcium phosphate porous microspheres	Physical entrapment<br>Adsorption	Strontium<br>Vancomycin	-<br>Higuchi release profile	[93]
Gelatin/collagen and bioactive glass fibers	Physical entrapment	Copper	-	[62]
Mg and CO_3 doped apatite	Physical entrapment	Strontium, Magnesium	Zero order release	[107]

pharmaceutics

Bioglass microspheres	Physical entrapment	Strontium, Zinc, Silicon	Burst release profile	[108]
Complex-shaped HAp scaffolds	Physical entrapment	Lysozyme, BSA	Burst release profile	[110]
Chitosan/HAp scaffolds	Covalent bonding	BMP2-derived peptide P24	Zero order release	[111]
electrospun poly (L-lactide)/HAp	Covalent bonding	Arg–Gly–Asp (RGD)	-	[113]
